# Analysis Of Salivary Herpesviruses Reveals Associations Between HHV-6 And Long COVID Severity

**DOI:** 10.64898/2026.05.19.26353495

**Published:** 2026-05-21

**Authors:** Claire S. Laxton, Alexandra Tabachnikova, Lily Cooke, Kexin Wang, Simone Blaser, Julio Silva, Jamie Wood, Henna S. Nam, Zhenni Lu, Christine Miller, Gisele Rodrigues, Victoria Fisher, Christian Guirgis, William B. Hooper, Alexandra Lee, Mackenzie Doerstling, Bornali Bhattacharjee, Leying Guan, David Putrino, Akiko Iwasaki

**Affiliations:** 1 Department of Immunobiology, Yale School of Medicine, New Haven, CT; 2 Department of Rehabilitation and Human Performance, Icahn School of Medicine at Mount Sinai, New York; 3 Department of Biostatistics, Yale School of Public Health, New Haven, CT; 4 Department of Medicine, Division of Infectious Diseases and Global Health, Yale School of Medicine, New Haven, CT; 5 Department of Pediatrics, Division of Infectious Diseases and Global Health, Yale School of Medicine New Haven, CT; 6 Center for Infection and Immunity, Yale School of Medicine, New Haven, CT; 7 Howard Hughes Medical Institute, Chevy Chase, Maryland

## Abstract

**Background:**

Reactivation of human herpesviruses (HHVs), particularly EBV, is associated with more severe acute SARS-CoV-2 infections and the development of Long COVID (LC). Observations of higher anti-EBV antibody levels in individuals with LC support the idea that chronic reactivation of HHVs could contribute to LC pathology. HHV shedding in saliva has also been previously associated with saliva hormone levels. This study aims to examine the relationship between salivary shedding of HHV DNA and LC symptoms, as well as cortisol, testosterone, and estradiol levels.

**Methods:**

We enrolled 45 participants with LC, and 45 age-sex-matched controls. Surveys and validated health questionnaires were used to collect demographics, medical history, and symptom profiles. Saliva was self-collected at waking, 15, 30, and 45 minutes, and 8 and 16 hours after waking, across two consecutive days. Salivary cortisol, testosterone and estradiol were measured, and extracted nucleic acid was tested for EBV, HSV 1/2, HCMV and HHV-6 A/B using multiplex qPCR, plus SARS-CoV-2 and RNaseP using RT-qPCR.

**Findings:**

Detection of salivary EBV and HHV-6 DNA was highest early in the morning. There were no significant differences in salivary cortisol, testosterone, or estradiol, or in EBV or HHV-6 shedding between the LC and control groups. However, salivary HHV-6 DNA levels were positively associated with a greater aggregated LC propensity score, as well as anxiety and depression scores.

**Interpretation:**

The observed correlation between salivary HHV-6 shedding and symptom severity suggests HHV-6 may contribute to post-acute disease, though mechanisms remain unclear. While our study did not identify a relationship between salivary EBV shedding and LC, EBV may still play a role at earlier time points in the disease course, or in compartments not sampled here. These findings highlight the potential importance of HHV-6 in LC pathophysiology and underscore the need for longitudinal, multi-compartment studies of herpesvirus reactivation in LC.

## Introduction

Herpesviruses are DNA viruses that establish lifelong latency in their hosts and reactivate in response to stressors ^1,2^. There are nine human herpesviruses (HHVs). Most individuals become infected with one or more HHV during early life, typically with a mild primary infection. All HHVs, except varicella zoster virus (VZV), are shed in saliva during both primary infection and reactivation, and this can occur without accompanying symptoms ^1^. Recently, HHV latency and reactivation have been associated with, or causally linked to, numerous chronic autoimmune disorders, including Multiple Sclerosis (MS) ^3-7^, Systemic Lupus Erythematosus (SLE) ^8,9^, Myalgic Encephalitis/Chronic Fatigue Syndrome (ME/CFS) ^10,11^, and other neurodegenerative diseases ^12-14^.

Almost as soon as SARS-CoV-2 emerged, HHVs, particularly EBV, have also been implicated in both increased acute COVID-19 disease severity ^15-19^ and the development of the post-acute SARS-CoV-2 syndrome known as Long COVID (LC) ^20-26^. Su *et al*. demonstrated that EBV viremia during acute SARS-CoV-2 infection was associated with fatigue, memory problems, and sputum production in those who subsequently developed LC ^21^. Studies have also demonstrated that people with LC have elevated anti-EBV antibodies, indicative of a recent reactivation ^20,23,26^. Rohrhofer *et al*. detected EBV DNA in the throat washes of people with LC an average of 235 days after acute infection, at significantly higher rates than in recovered controls (50% vs 20%), although they did not observe differences in EBV-specific antibodies ^24^. Additionally, Zubchenko *et al*. demonstrated that individuals with LC who tested positive for reactivation of EBV and/or HHV-6 by PCR were more likely to experience sleep disorders, joint pain, hair loss and pulmonary symptoms ^22^. The authors did not distinguish the effects of EBV and HHV-6, nor HHV DNA detection in blood vs saliva, and thus, the precise relationships between observed elevations in oral herpesvirus DNA levels and LC symptoms remain unclear.

Previous studies have also identified altered hormonal signaling in LC, including decreased blood cortisol levels ^23^, particularly during early morning timepoints ^27^. Moreover, LC is more prevalent in women, and differences in sex hormone levels have been associated with immunological changes in sex-segregated analyses of LC ^28-30^. However, prior studies looking at hormone levels in LC have used serum samples from a single timepoint.

Collection of salivary specimens presents an opportunity to robustly interrogate diurnal expression of stress and sex hormones ^31-38^, as well as HHV reactivation ^1^, as it is non-invasive and simple to self-collect over many timepoints ^39^. This study aimed to intensively characterize the salivary shedding dynamics of EBV, HSV, HCMV and HHV-6 genomic DNA levels, and explore their associations with hormones and LC symptoms, to better establish the incidence and impact of chronic herpesvirus reactivation in people with LC.

## Methods

### Ethics Statement

This study was approved by the Mount Sinai Program for the Protection of Human Subjects (IRB 20-01758) and the Yale Institutional Review Board (IRB 2000029451). Written informed consent was obtained from all enrolled participants.

### Study design

This was a satellite study of the MY-LC (Mount Sinai - Yale Long COVID) project, a cross-sectional multi-site retrospective case-control study examining LC ^23^. This sub-study, which is reported in accordance with the STROBE guidelines for observational studies, was a repeated-measures, cross-sectional, case-control study in which participants were sampled over two consecutive days during the period of April 2023 to September 2024, with no further study visits. Participants were recruited from the New York City area, as described in Supplementary Methods.

LC cases were included if they were aged ≥18 years old and were diagnosed with LC, defined as having symptoms persistent >6 weeks following initial COVID-19 infection. Convalescent controls were included if they were aged ≥18 years old and reported either no previous COVID-19 infection or a complete recovery from COVID-19 within 6 weeks. Participants were excluded if they could not provide informed consent and had conditions preventing saliva collection. Post-enrollment, based on the subsequently available 2024 NASEM LC Definition ^40^, the LC case definition use was refined; cases were excluded if their initial SARS-CoV-2 infection was <3 months before enrollment and controls were excluded if they reported any SARS-CoV-2 infection <3 months before enrollment. Participants were also excluded if they reported a current or previous diagnosis of an autoimmune disease or immune deficiency, usage of any immunosuppressive or anti-herpes antiviral medication in the last 3 month, or if they did not meet age-sex matching criteria (matched reported sex and age, ±5 years).

### Participant surveys

One day prior to sample collection, demographic information and self-reported medical histories were collected using online surveys and further reviewed through examination of the electronic medical records (EMR) of collaborating clinics, or individual follow up, where required. Participants provided their gender on surveys, and biological sex was confirmed by EMR review or individual follow up, and in all cases, gender and sex matched, and sex is reported hereon.

At the time of the initial survey, participants also completed the following validated health questionnaires: EuroQol 5-Dimension 5-Level health related quality of life (EQ-5D-5L) ^41^, Medical Research Council (MRC) dyspnea scale (MRC dyspnea) ^42^ , Fatigue Severity Scale (FSS-7) ^43,44^, Patient Health Questionnaire (PHQ-2) ^45^, Generalized Anxiety Disorder 7 (GAD-7)^46^, Self-report Leeds Assessment of Neuropathic Symptoms and Signs pain scale (S-LANSS) ^47,^ Epworth sleepiness scale (ESS) ^48^, Single-Item Sleep Quality Scale (SQS) ^49^, Adult PROMIS Ability to Participate in Social Roles and Activities short form 8a (PROMIS Participation) ^50,51^, Neuro-QoL Adult Cognitive Function short form (NQ-Cognitive) ^52,53^, DePaul Symptom Questionnaire - Post-Exertional Malaise short form (DSQ-PEM) ^54^, and General Symptom Questionnaire-30 (GSQ-30) ^55^. Additionally, participants scored their overall health on that day (eq5), and global fatigue and breathlessness over the past week using 0–100-point visual analog scales (VAS).

Additionally, at the time of each saliva sample collection, participants were asked to fill in a short online survey, detailed in Supplementary Figure 1. Here, participants were asked to identify the worst symptom they were experiencing in that moment, using a free-text answer. These answers were encoded into categories according to the end organ/body system affected as described in Supplementary Methods.

Survey data was collected using REDCap and stored on Icahn School of Medicine, Mount Sinai servers. Once all data was collected, a copy of the de-identified database was transferred to researchers at Yale for analysis. Raw data from validated health questionnaires were aggregated and normalized according to their respective protocols.

### Sample collection and processing

Participants provided 12 saliva samples, 6 samples per day, self-collected at home by passive drooling into a 5 mL sterile Eppendorf tube. The collection schedule and restrictions are shown in Supplementary Figure 1. Samples were stored in home refrigerators then shipped by courier within 120 hours to Icahn School of Medicine, where they were stored at −80°C. Samples were then shipped to Yale School of Medicine on dry ice and stored at −80°C.

At Yale, de-identified saliva specimens were inventoried and managed using the unique alphanumeric codes assigned at the time of collection. Saliva samples from each participant were thawed at the same time on ice. Saliva was centrifuged at 2500 xg for 15 minutes at 4 ° C. Supernatants were aspirated and aliquoted under enhanced BSL-2 conditions, then stored together with remaining pellets at −80 ° C. For all downstream testing, as far as possible, matched pairs were run in the same batches.

### Salivary hormone quantification

Aliquots of saliva supernatant were shipped to Salimetrics, LLC (Carlsbad, CA, USA) on dry ice and analytes were measured for cortisol (5100), testosterone (5138), and estradiol (5160) by ELISA in duplicate and then averaged. Cortisol and testosterone analysis was conducted in three batches with internal controls in each shipment to correct for kit batch effects. Details on assay sensitivity, range and coefficients of variation, as well as the methods used for batch correction can be found in Supplementary Methods.

Diurnal cortisol and testosterone analyses were conducted on natural-log-transformed values to reduce the right-skewness of the data distribution and mitigate the impact of outliers. For the Area Under the Curve (AUC) and Cortisol Awakening Response (CAR) AUC, individual natural log-transformed values for two consecutive days of each time point were averaged and then AUC was calculated. For participants without 2 samples per timepoint, the single timepoint was used in place of the average. AUC measures were calculated using the trapezoid method, with respect to ground and the waking timepoint for Whole Day AUC and CAR AUC, respectively, as described previously ^59^. For Waking testosterone, values were averaged between the first timepoint of two consecutive days. For Estradiol, a single morning timepoint value was measured.

### Salivary herpesvirus DNA quantification

As far as possible, 6 samples for each participant were tested: timepoints 0 (immediately after waking), 8 and 16 hrs after waking, from both days. Where a timepoint 0 sample was unavailable, for example due to insufficient volume, the next earliest morning sample available (e.g. 15-45 min after waking) was used instead.

#### DNA extraction

Aliquots of 200 μL salivary supernatant were thawed in batches on ice and briefly spun to collect the liquid at the bottom of the tubes. Where sample volume was insufficient, the actual extracted volume was noted. As far as possible, total nucleic acid (TNA) was extracted from 175 μL of each sample using the MagMax Viral Pathogens Il kit and KingFisher Apex as described previously ^60^, except using the MVP-II protocol, and including two elution steps, each into 50 μL elution buffer. Each 96-well extraction plate also included 6 negative extraction controls (nuclease-free water) interspersed throughout. Following elution, samples were immediately transferred to tubes and stored at −80 ° C.

#### qPCR and RT-qPCR

The two assays, a multiplex qPCR to detect gDNA from the herpesviruses EBV, HSV 1 and 2, CMV and HHV-6 A and B, and a dualplex Reverse-Transcriptase (RT) qPCR to detect RNA from SARS-CoV-2 and total nucleic acid from human RNAseP, were run simultaneously on each batch of samples, as far as possible. Primers and probes for each assay were obtained from Integrated DNA Technologies (IDT, USA), and are listed, along with the final concentrations used, in Supplementary Table 1. Both assays used 5 μL template per reaction, run in duplicate, in a final volume of 20 μL. Duplicate no-template control (NTC) wells using nuclease-free water were included on every plate.

The herpesvirus multiplex used qPCR, Luna^®^ Universal Probe qPCR Master Mix (New England Biolabs, USA), with the following conditions: 95 ° C for 2 mins, (95 ° C for 15 secs, 60 ° C for 30 secs) * 40 cycles. For standard curves, the following quantitative herpesvirus gDNA standards were mixed 1:1:1:1 to make ten-fold dilutions ranging from 50,000-5 copies/μL: VR-3247SD, VR-538DQ, VR-1493DQ, VR-1467DQ (American Type Culture Collection, USA). The efficiency and Lower limit of quantification (LLOQ) for each target in the multiplex qPCR was validated using standard curve analysis, as described in detail in Supplementary Methods. An individual was considered positive if a given virus was detected in ≥2 of their samples above the LLOQ.

For the SARS-CoV-2/RNASeP RT-qPCR, the Luna^®^ Probe One-Step RT-qPCR Kit (No ROX) was used (New England Biolabs, USA), with the following conditions: 55 ° C for 10 mins, 95 ° C for 2min, (95 ° C for 30 secs, 55 ° C for 30 secs) * 45 cycles. The following positive controls were used at a single concentration: Hs RPP30 positive control (IDT, USA) at 5,000 copies/μL and the Assay Ready Synthetic SARS-CoV-2 RNA Control (Twist Biosciences, USA) at 100 copies/μL. An individual was considered positive if SARS-CoV-2 amplified with a Cq <40 in ≥1 of their samples.

Thermocycling was done in two side-by-side BioRad CFX96 Touch machines, within the same calibration cycle. Following fluorescence baseline correction and manual inspection of the curves, data were exported as CSV files. A custom Python3 extract-transform-load pipeline was created in-house for qPCR data analysis. This pipeline allows for rapid compilation and QC of multiple raw qPCR data files into one clean data sheet, ready for downstream analysis and is described in Supplementary Methods. Where the pipeline marked samples ‘Inconclusive’, these were re-run a maximum of twice, and those that remained Inconclusive were excluded from analysis. Viral copies were converted to total copies/input in the reaction by multiplying by 5.^57^

### Statistical analysis

Statistical significance of differences in hormone levels and viral copies between LC and control groups was assessed using Wilcoxon rank-sum test or signed-rank test, as indicated, as well as by regression modelling, described below. Spearman correlations between herpesvirus copies and hormones were also performed. Where indicated, multiple testing was corrected using a Benjamini-Hochberg (BH) correction, with a False Discovery Rate of 5%.

### Hierarchical symptom clustering

To assess whether participants cluster by symptom burden measured by the GSQ-30, we performed hierarchical clustering on the symptom severity profiles. Participant–participant dissimilarity was computed using Gower distance, treating symptoms as ordinal severity measures, while symptom–symptom distance was calculated from Spearman correlation. We then applied hierarchical clustering with average linkage to both the participant and symptom dimensions. The number of participant clusters was selected using the gap statistic: we chose k=3 because the gap increased sharply from k=2 to k=3, with only marginal gains for k>3 (Supplementary Figure 3A).

### Long COVID propensity score composition

We constructed a LC propensity score (LCPS) using a LASSO logistic regression model from the following 14 survey-based measures: EQ-5D-5L USA-indexed score, FSS-7 mean, PHQ-2 sum, GAD-7 sum, ESS sum, PROMIS Participation T-score, NQ-Cognitive T-score, DSQ-PEM sum, MRC dyspnea, SQS, GSQ-30 sum, eq5 VAS, Fatigue VAS, Breathlessness VAS. This score followed the same conceptual framework as reported previously^23^. All survey variables were standardized with LC status as the binary outcome. Predictive performance was evaluated using nested cross-validation, with 10 outer folds for performance estimation and 10 inner folds for model tuning, summarized by AUROC (Figure 2D). We then refit the model on the full dataset and selected the final penalty parameter using the one-standard-error rule to favor a parsimonious solution. The surveys with non-zero coefficients in the final model are shown in Supplementary Figure 3B. For each participant, we then derived the model-based linear predictor as the LCPS, where higher values indicate survey profiles more strongly aligned with LC. We assessed the association between LCPS and HHV-6/EBV and salivary hormone levels using Spearman correlation and linear regression models adjusted for age, sex, and BMI with BH adjusted p-values.

### Modelling EBV and HHV-6 salivary DNA detection across time

We analyzed longitudinal viral measurements using a Bayesian mixed-effects model. The 2-day average for each timepoint and participant was taken. Values below the lower limit of quantification (LLOQ = 3) were treated as left-censored at LLOQ. We then fit a Bayesian lognormal mixed-effects model using R package brms ^61^ with fixed effects for LC group, age, sex, BMI, and categorical time points, along with a participant random intercept, and we reported posterior means and 95% credible intervals.

### Regression modelling of symptoms, viral copies and hormones

Replicate virus measurements were averaged within each participant at the same time point across the two study days. We used linear mixed-effects models (lmer in R package lme4 ^62^) to account for repeated measures per participant. We reported all regression coefficients with 95% confidence intervals and highlighted associations with a nominal p<0.05. Within each model, BH correction was applied to all EBV and HHV-6 p-values.

Fatigue was modeled as the outcome, with fixed effects for categorical relative time to the first measurement, LC group, HHV-6, EBV, age, sex, and BMI, and a random intercept for participant ID. We then fit the same model separately within the LC and control subgroups to estimate within-group associations.

For each salivary hormone outcome, we fit linear regression models with mean log (HHV-6 or EBV +1) as predictors, adjusting for age, sex, BMI, hormonal medication use, and time since first SARS-CoV-2 infection (binned as <2 years or >2 years); in the combined cohort we additionally adjusted for LC versus control group. We also fit stratified models separately in LC and control participants as well as by sex.

For each validated health questionnaire outcome, we fit linear regression models with mean log (HHV-6 or EBV +1) as predictors, adjusting for age, sex, and BMI; in the combined cohort we additionally adjusted for LC versus control group. We also fit stratified models separately in LC and control participants. Due to their inclusion in the LCPS analysis, the three VAS questions (fatigue, breathlessness and general health-eq5) were also included in these analyses.

## Results

### Participants with Long COVID report an overall high disease burden across multiple metrics

**Figure 1 :**
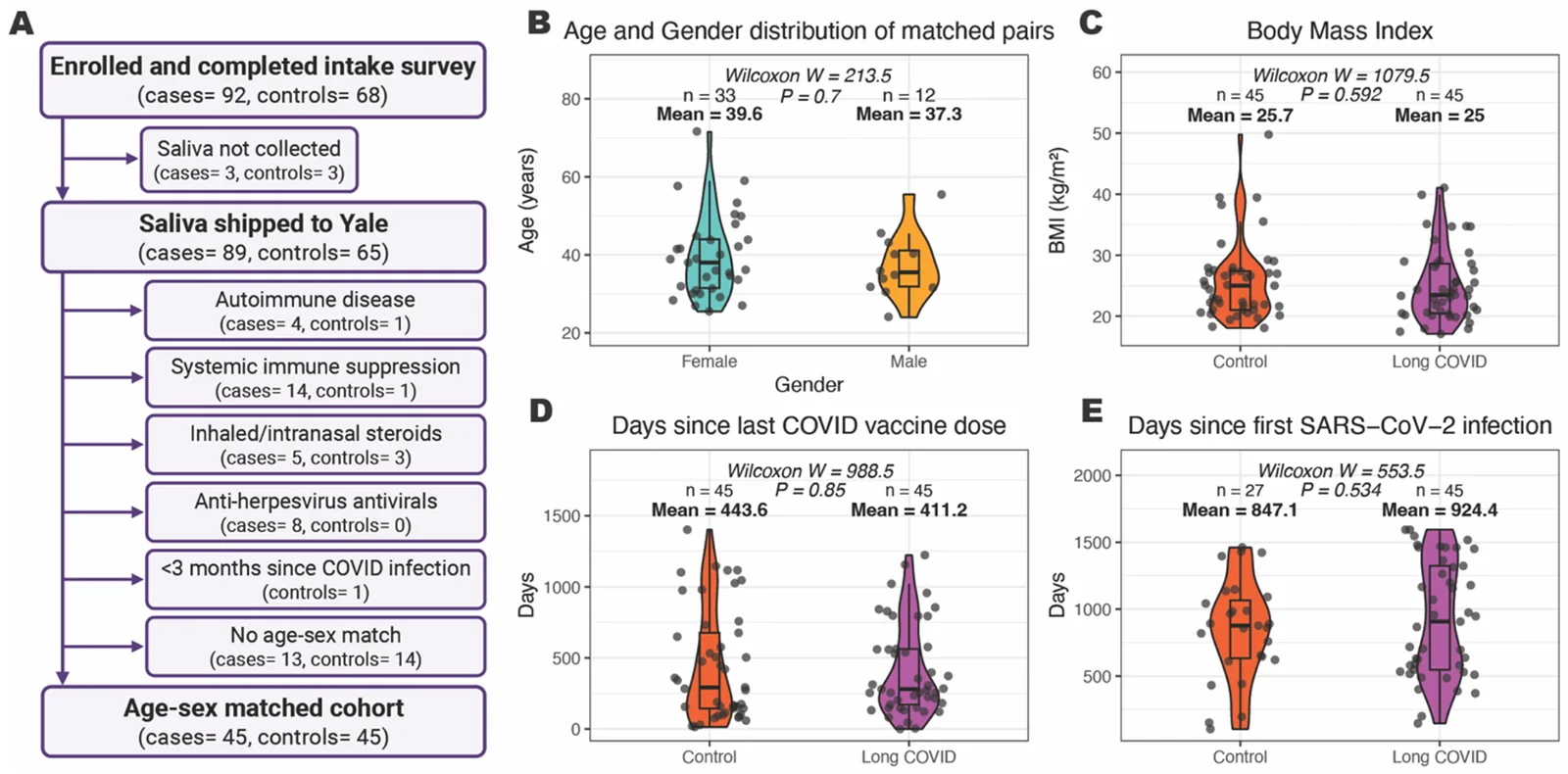
Cohort description and demographics. **A.** Flow chart of participant inclusion in the study. Cases defined as people self-reporting a Long COVID diagnosis, Controls are defined as those who reported never experiencing or being fully recovered from COVID-19. **B-E.** Description of MY-LC Saliva cohort characteristics. Differences between groups determined by Wilcoxon test, p<0.05 considered significant.

In total, 160 participants were enrolled in the study (92 LC, 68 Controls) and following post-enrollment exclusions, as summarized in Figure 1A, 90 participants (45 age-sex matched pairs) were included for our analyses. Participants’ demographics, self-reported medical history, and medication usage are shown in Figure 1B-E and Supplementary Table 2. There were no statistical differences in BMI, days since last COVID vaccine dose, or days since last SARS-CoV-2 infection (Figure 1B-E). As expected, prior COVID-19 incidence, levels of medication use, and co-morbidities were higher in the LC group.

Validated health questionnaire outcomes are summarized in Table 1. Between-group comparisons, using paired Wilcoxon signed-rank tests, revealed significant differences; all the following findings had a BH-adjusted p-value <0.001. Overall, compared with convalescent controls, those with LC reported severely reduced quality of life (EQ-5D-5L r= −0.87), cognitive function (NQ-Cognitive r= −0.85), and ability to participate in daily activities (PROMIS Participation r= −0.86) as well as more severe fatigue (FSS-7 r= 0.79), post-exertional malaise (DSQ-PEM r= 0.87), breathlessness (MRC dyspnea r=−0.62) and general symptom burden (GSQ-30 r= 0.87). Additionally, those with LC reported moderately reduced sleep quality (SQS r=0.38) and increased daytime sleepiness (ESS r= 0.36), as well as moderately increased symptoms of anxiety (GAD-7 r= 0.52), and slightly increased symptoms of depression (PHQ-2, r= 0.19).

**Figure 2:**
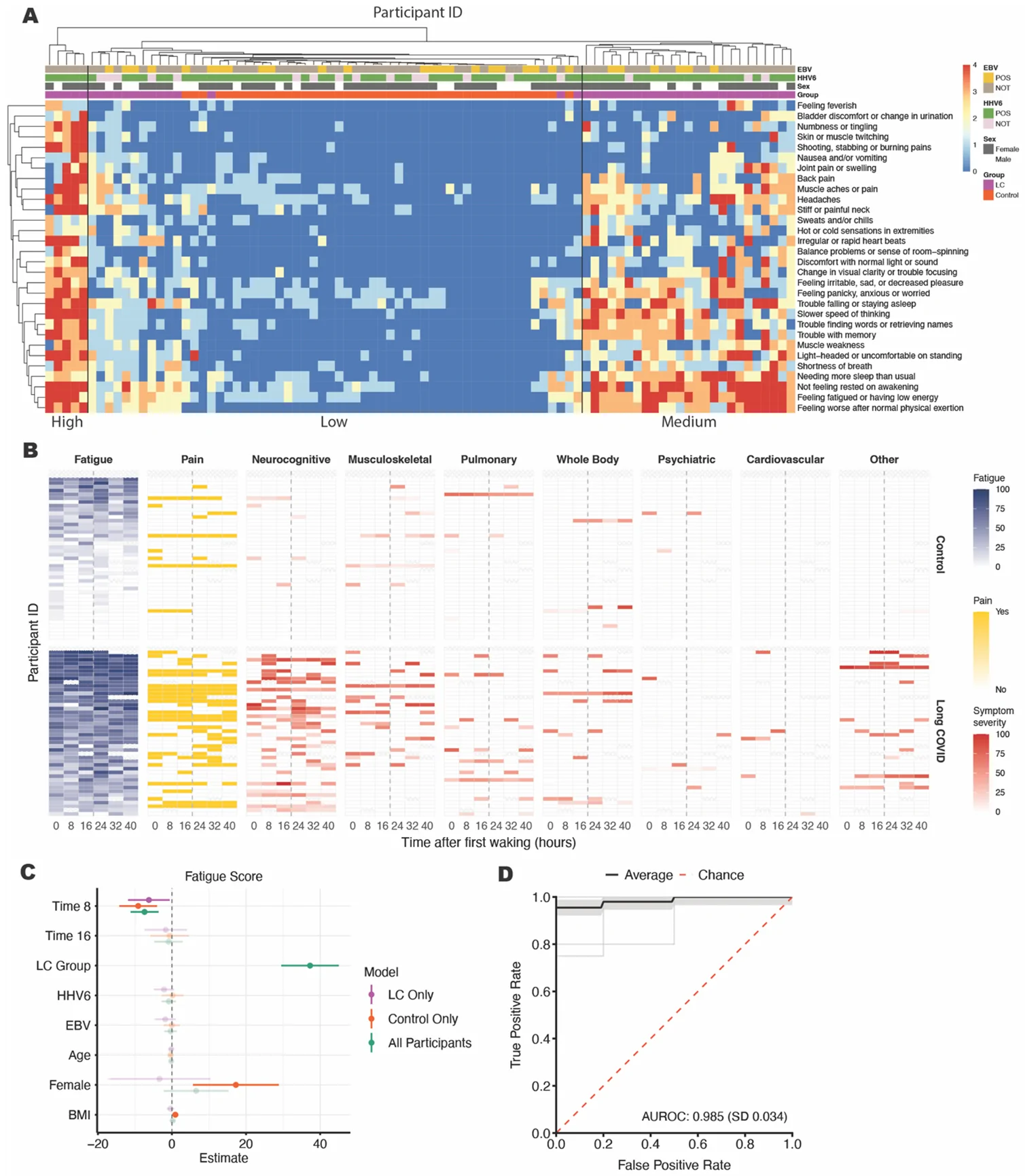
Analysis of symptom survey responses. **A.** Hierarchical clustering analysis of General Symptom Questionnaire (GSQ-30), using Gower distances (LC= Long COVID). EBV and HHV-6 positivity and sex are also indicated. n = 88 (44 LC, 44 control). **B.** Heat map showing self-reported fatigue, pain and the worst reported symptom, encoded by end-organ-system, at each sample collection timepoint. **C.** Linear mixed-effects models, modelling fatigue at a given time point (as in panel **B)**, with fixed effects for categorical relative time to 0 hours after waking, LC group, HHV-6, EBV, age, sex, and BMI, and a random intercept for participant ID. Models were fit within LC and Control subgroups. Regression coefficients are reported with 95% confidence intervals, and those in bold are associations with p<0.05. **D.** Receiver Operating Characteristics (ROC) curve to assess predictive performance of the LASSO model to generate the Long COVID Propensity Score (LCPS).

Hierarchical clustering analysis of the GSQ-30 data using Gower distance, shown in Figure 2A, revealed that symptom severity clustered into low (n=45 Controls, n=14 LC), medium (n=25 LC) and high (n=5 LC) burden groups. At the time of each sample collection, participants rated their fatigue and recorded the single worst symptom they were experiencing, as well as its severity using a 0-100 VAS. These symptoms were encoded into end-organ-system categories and further classified according to whether pain was a feature (Figure 2B). Neurocognitive symptoms were most frequently reported, primarily headache and brain fog (Figure 2O), followed by musculoskeletal and pulmonary symptoms. Those with LC reported significantly higher levels of fatigue (Figure 2C). Overall, participants also reported their lowest fatigue during the day, 8 hours after waking (0 vs. 8 hours post waking, p= <0.001, 16 vs. 8 hours post waking, p= <0.001). When stratified by LC status, in the control group only, higher fatigue was associated with being female (p= 0.005) and having a higher BMI (p= 0.028).

To produce a quantifiable metric of symptom magnitude and prevalence, survey responses were compiled to produce a single metric, the Long COVID Propensity Score (LCPS). This score was generated using a LASSO-penalized logistic regression model, following the same conceptual framework previously described for MY-LC study participants^23^. The LCPS performed remarkably well in differentiating LC participants from Controls (AUC = 0.985, SD = 0.034, Figure 2D). The overall LCPS disease severity for each symptom cluster defined in Figure 2A is shown in Supplementary Figure 3C.

### There were no differences in salivary cortisol, testosterone or estradiol between Long COVID and control groups

Approximately 20% (n=19) of participants, all female, reported taking hormonal medication. Hormonal contraceptives, including hormonal intrauterine devices, were the most reported hormonal medication, and hormonal medication use was balanced across LC and Control groups (Supplementary Table 2). Additionally, 4 individuals self-reported hormonal disorders, hyperthyroidism (n= 1), polycystic ovary syndrome (PCOS, n=2) and hypothyroidism plus PCOS (n=1). Therefore, for our hormone analyses, we excluded these 4 participants with hormonal disorders and adjusted our linear models for hormonal medication use.

**Figure 3:**
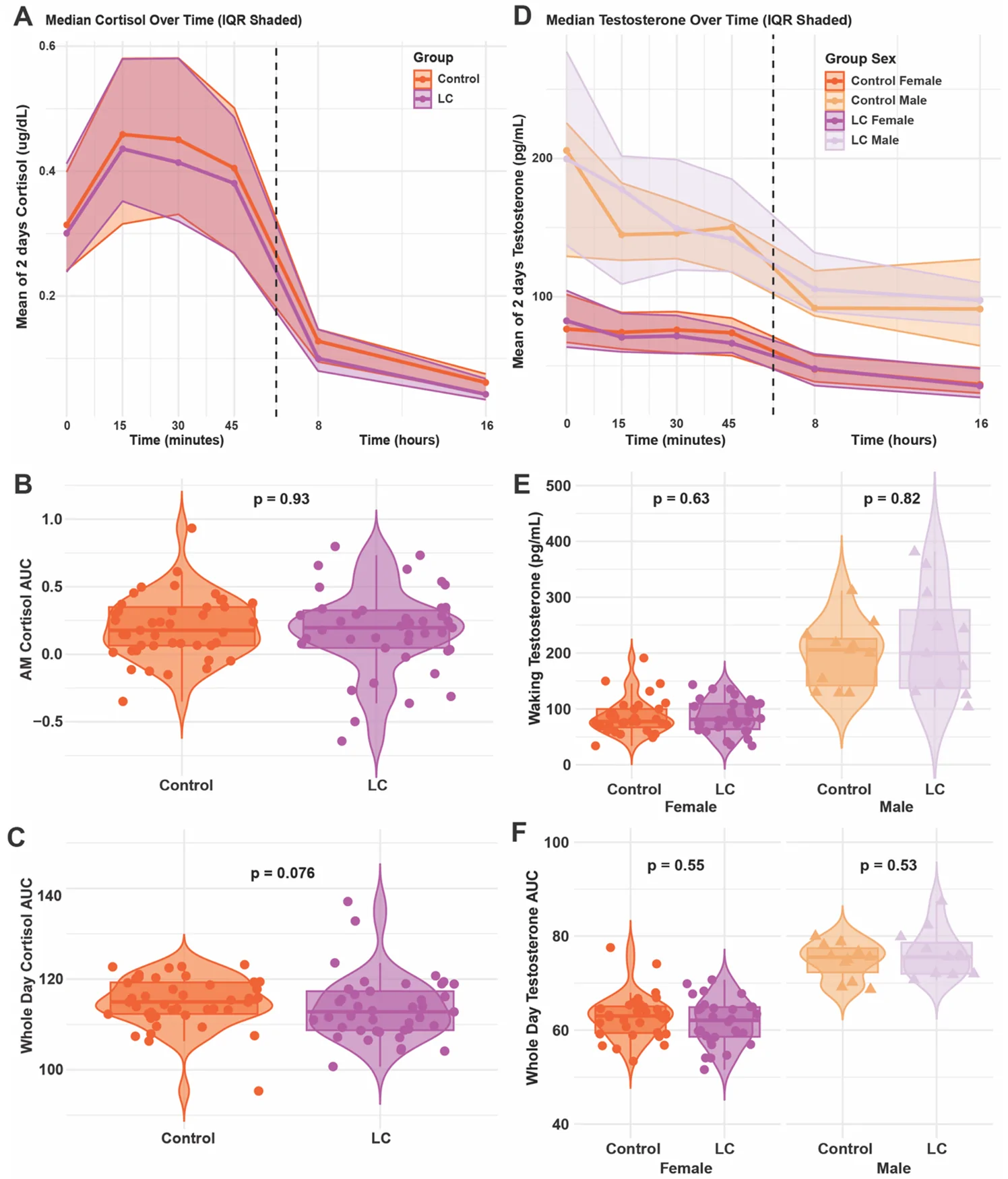
Salivary Hormones are not significantly different in Long COVID. **A, D.** Line plot showing median and Interquartile Range of 2-day mean of salivary Cortisol (**A**) and Cortisol (**D**) for each timepoint by group. Dashed vertical line indicates split of morning cortisol from afternoon and evening. **B, C.** Comparison of Area Under the Curve of Cortisol Awakening Responses for the first 4 timepoints, (**B**) and Area Under the Curve of Whole Day Cortisol for 6 timepoints, (**C**), averaged over 2 days by group. n=43 (LC) and n=43 (Control). **E, F.** Comparison of 2-day average of Waking Testosterone at the first timepoint (**E**) and Area Under the Curve of Whole Day Testosterone for 6 timepoints, averaged over 2 days, (**F**), by group. n=30 (LC Female), n=11 (LC Male), n=32 (Control Female), and n=11 (Control Male). For the violin boxplots, central lines indicate the group median values, and the top and bottom lines indicate the 75^th^ and 25^th^ percentiles, respectively. The whiskers represent 1.5 x the interquartile range. Each dot represents one individual. Statistical significance of the difference in median values was determined using Wilcoxon ranked-sum tests.

To capture diurnal dynamics of hormone production, salivary cortisol and testosterone were measured at 4 morning time points (in 15-minute increments from waking), an afternoon time point (8 hours after waking), and an evening time point (16 hours after waking) over two consecutive days using ELISA. Salivary estradiol was measured at a single morning time point. Cortisol peaked 15-30 minutes after waking and was secreted at similar levels in participants with LC and controls (Figure 3A). To quantify total Cortisol Awakening Response (CAR), AUC was measured from waking levels to 45 minutes, and it did not differ between LC and Control (Figure 3B). Total cortisol production over the course of the day measured with AUC trended lower in LC than Control but did not reach significance (p= 0.076, Figure 3C). Morning and whole day cortisol was not associated with LC status, herpes viral shedding, sex, BMI, hormonal medication use, or time from infection when assessed using a linear model (Supplementary Figure 4A-B).

Testosterone, too, showed a diurnal production pattern, peaking upon waking and decreasing throughout the day, with higher levels in males (Figure 3D). However, testosterone was produced at similar levels in LC and Controls, both at waking (Figure 3E) and throughout the whole day as measured with AUC (Figure 3F). In both groups, males produced significantly more testosterone than females (p <0.001, Figure 3E-F). Similarly, morning estradiol levels did not differ by group, but had a greater range of production in females, likely reflecting variations related to menstrual cycle (Supplementary Figure 4F).

When analyzed using a linear model, sex hormone levels were not associated with LC status, when adjusted for age, sex, BMI, time from infection and hormonal medication use (Supplementary Figure 4A-E). When stratified by sex, there were no significant associations between hormone level and LC status (Supplementary Figure 4G-K), though there was a small, non-significant trend toward lower whole-day testosterone in the Female-only LC group (p= 0.080, **Supplementary Data 1**). Hormone levels also did not correlate with LCPS (Supplementary Figure 4L-O).

### Shedding of neither EBV nor HHV-6 DNA was singularly predictive of Long COVID status

**Figure 4:**
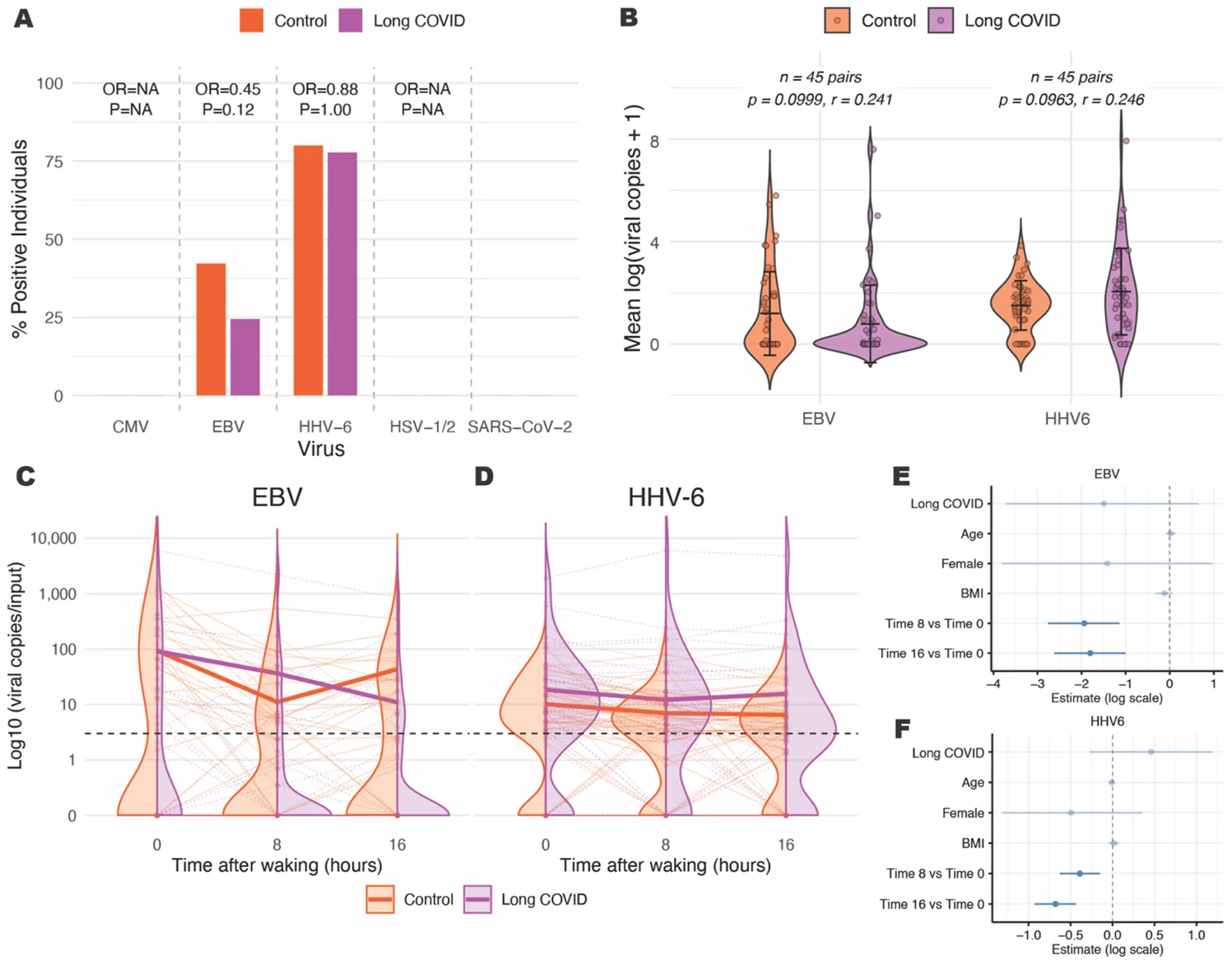
Detection of salivary herpesviruses. **A.** Positivity rate of all participants for each virus. Statistical differences tested using Fisher’s Exact test, OR= Odd’s Ratio, P<0.05 is significant. **B.** Comparison of mean log (viral copies+1) between participants with Long COVID and controls. Mean and SD are shown. Statistical differences tested using Wilcoxon signed-rank test, effect size based on the mean difference, p<0.05 is significant. **C-D.** Split violin plots showing viral copies of EBV and HHV-6 detected for each timepoint, averaged from 2 days. Bold line shows median of all samples >LLOQ (dashed black line). **E-F.** Forest plots from a Bayesian left-censored log-normal mixed model, showing associations between EBV and HHV-6 mean log (viral copies) +1, respectively, with timepoint and Long COVID status, adjusted for age, sex and BMI. Posterior mean and 95% credible intervals are reported, bold lines are those with credible intervals that do not cross zero.

Saliva samples were tested for EBV, HSV 1 and 2, CMV, HHV-6 A and B DNA by qPCR, and SARS-CoV-2 RNA by RT-qPCR. An average of 6.0 samples per Control and 6.2 samples per LC participant were tested, from 0, 8, and 16 hours after waking over two consecutive days. EBV and HHV-6 positivity rates in Controls were 42.2% and 80.0%, respectively, and 24.4% and 77.8%, respectively in LC participants (Figure 4A). Co-presence of EBV and HHV-6 was observed in 40% of Control participants and 20% of LC participants. Additionally, no participants were positive for SARS-CoV-2 RNA, or HCMV or HSV-1/2 DNA.

There were no significant differences in viral positivity (Figure 4A) or proportion of positive samples per participants between groups (Supplementary Figure 5A). Similarly, although there was a trend toward higher mean HHV-6 DNA (r= 0.246, p=0.096) and lower mean EBV DNA (r=−0.241, p= 0.099) copies in the LC group, the differences were not statistically significant (Figure 4B). There were no differences in area under the curve across all time points for either virus (Supplementary Figure 5B). Additionally, EBV and HHV-6 viral copies were not significantly correlated with each other (Supplementary Figure 5C).

Two-day salivary shedding of EBV and HHV-6 DNA for each participant is shown in Figure 4C-D. Analysis using a Bayesian left-censored log-normal mixed model found that detection of higher levels of EBV and, to a lesser extent HHV-6, DNA was more likely at the first timepoint of the day compared to later timepoints (Figure 4E-F). This model also supported the non-significant trends in viral copies and Long COVID status.

### Higher salivary HHV-6 DNA was associated with worse overall Long COVID symptom severity, anxiety, and depression

**Figure 5:**
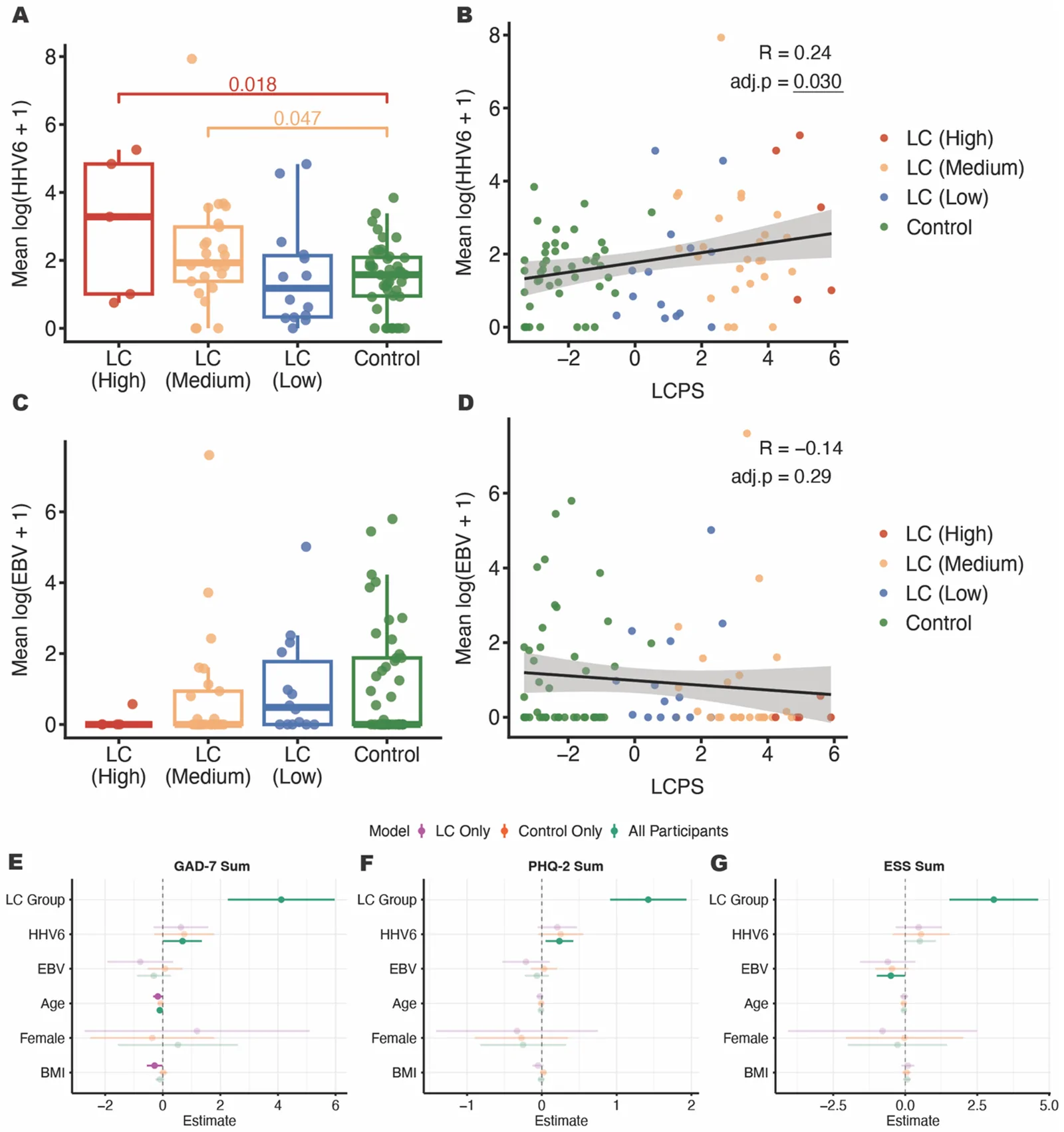
Relationships between salivary herpes DNA detection and Long COVID symptom severity. **A, C.** Boxplot showing HHV-6 (**A**) or EBV (**C**) DNA copies split according to GSQ-30 symptom cluster. differences were tested using linear regression model with Control as the reference, adjusted for age, sex and BMI, nominal p<0.05 is significant, n = 88 (44 LC, 44 control). **B, D.** Correlation analysis between Long COVID propensity score and HHV-6 (**B**) and EBV (**D**) DNA copies, Spearman coefficients (R) are shown, significant determined by linear regression adjusting age, sex and BMI, Benjamini-Hochberg False Discovery Rate (FDR) adjusted (adj.) p <0.05 was considered significant, n = 88 (44 LC, 44 control). **E-G.** Forest plots showing the association between anxiety (GAD-7, **E**), depression (PHQ-2, **F**) and daytime sleepiness (ESS, **G**) scores and Long COVID status, EBV and HHV-6 mean log (viral copies) +1, adjusted for age, sex and BMI, and stratified by Long COVID status. Regression coefficients are reported with 95% confidence intervals and those in bold are associations with a nominal p<0.05.

Next, we examined associations between salivary viral DNA and LC symptoms. When comparing the GSQ-30 clusters derived from Figure 2A, HHV-6 differed across groups (one-way ANOVA, p = 0.0402). After adjusting for demographics, those with LC in the High and Medium severity groups had higher salivary HHV-6 DNA copy numbers than controls (Figure 5A, p = 0.018 and p = 0.047, respectively) though these did not survive multiple testing correction (**Supplementary Data 1**). Among those with LC, when analyzed using a linear model with LC severity clusters as ordered levels, HHV-6 showed a positive monotonic trend across ordered LC clusters (Low → Medium → High), although the linear trend did not reach significance (p = 0.075), likely due to limited sample size within LC clusters.

We then examined the relationship between salivary viral DNA copies and LCPS score. We found that among all participants, higher salivary HHV-6 DNA copy numbers were positively correlated with higher (i.e., worse) LCPS (Figure 5B, R=0.248, adj.p =0.030). However, there were neither significant differences in salivary EBV DNA copies between GSQ-30 symptom cluster group (Figure 5C), nor any significant correlations between LCPS and levels of salivary EBV DNA (Figure 5D).

To examine the relationships between salivary viral DNA shedding and individual symptom types, we used a linear model to conduct exploratory analysis of the effect of salivary HHV-6 and EBV DNA copies on health questionnaire outcomes. Higher HHV-6 copies were associated with increased GAD-7 (anxiety, p= 0.045) and PHQ-2 (depression, p= 0.013) scores in all participants (Figure 5E-F), and higher EBV copies were associated with lower ESS (daytime sleepiness, p=0.045) in all participants (Figure 5G). However, these effects did not survive multiple testing correction, and further research is needed to be conclusive.

Finally, we examined the associations between salivary hormones and either EBV or HHV-6 shedding. Those with higher waking testosterone were more likely to have higher levels of EBV DNA in their saliva, which was predominantly driven by males in the LC group (Supplementary Figure 6C, waking testosterone, *R*= 0.32, p= 0.041). This was supported by results from linear regression models which found a positive association between EBV and waking testosterone in all participants (p= 0.015) and in the LC-only model (p= 0.029) , (Supplementary Figure 4B) and in males only when stratified by sex (p= 0.049, Supplementary Figure 4H). However, these findings, both from the models and correlations, did not survive multiple testing correction.

## Discussion

This study aimed to characterize the relationship between herpesvirus reactivation, salivary hormones, and LC symptoms in patients ~2 years after initial SARS-CoV-2 infection. We observed that those with LC reported significantly worse symptoms, across a wide range of metrics.

The levels of salivary hormones we observed align with those observed previously ^63-68^. However, there were no significant relationships between salivary cortisol, testosterone or estradiol and LC status or LCPS. Although we observed a trend towards lower whole-day salivary cortisol in those with LC, this was not statistically significant. These observations contrast with previous findings by Klein et al. of lower serum cortisol in people with LC ^23^. The discrepancy may be explained by differences in measurement of free vs. bound hormone between serum and saliva ^33,35^, the viral variant that triggered LC, vaccination status, or by the fact that participants were enrolled ~2 years after their initial infection in this study, a much 15 later time from infection than Klein et al. Although, a lack of correlation between LC and hormones has previously been reported in patients ^28^ and in a post-COVID mouse model ^69^.

We observed an overall salivary viral DNA positivity rate of 33% for EBV and 79% for HHV-6 across the study cohort, in line with observations from control groups in previous studies ^70-74^. However, other studies report lower rates of HHV-6 positivity in saliva ^75,76^, and EBV detection rates of up to 60% ^76^, suggesting a high level of variability in the literature, likely driven by differences in methodology, geography, and shedding kinetics. No individuals in our study were positive for SARS-CoV-2, HCMV or HSV-1/2 during sample collection.

Additionally, we observed that salivary detection of EBV, and to a lesser extent, HHV-6 DNA was significantly higher immediately after waking compared with later timepoints. This has been observed previously for EBV ^77^ but not for HHV-6 to our knowledge. This observation may be explained by saliva from early timepoints being more concentrated due to accumulation during sleep. However, we adjusted for host DNA/RNA quantity, suggesting that higher shedding in the morning may be independent of sample ‘richness’ ^78^.

Previous studies have reported elevated antibodies against lytic antigens of EBV ^20,23^ in patients with LC, and EBV and/or HHV-6 positivity at the post-acute stage was associated with LC symptoms ^22^, suggesting that recent or ongoing reactivation may be driving disease. In this study, we observed no significant relationship between the levels of either HHV-6 or EBV DNA in saliva and LC status. These findings suggest that elevated antibodies against EBV likely reflect reactivation of EBV earlier in the development of LC, as has been described in the blood ^21^ and in the nasal mucosa ^18^ during acute infection, though longitudinal investigation of viral production and circulating antibodies is required to determine this relationship. Interestingly, we observed that higher waking salivary testosterone levels, particularly in males, were nominally associated with greater EBV shedding. This aligns with the observation that males have higher anti-EBV IgA titers, consistent with more frequent mucosal reactivation, while females have higher anti-EBV IgG ^79^ titers, a pattern previously observed in LC ^29^.

However, comparing a binary outcome- LC or Control - is somewhat crude given the heterogeneity of LC manifestations. In reality, participants’ symptomology fell on a spectrum, with overlap between Controls and those in the ‘low’ LC symptom burden group (Figure 2A).

To account for this, we created the LCPS score as a continuous measure of LC severity. Here, we observed that having higher levels of salivary HHV-6, but not EBV, DNA was correlated with a higher LCPS, and a greater symptom burden for those in the medium-to-high LC severity groups. Zubchenko et al. (2022) also observed a positive association between EBV and HHV-6 shedding and Long COVID symptoms, although they did not stratify their analysis by virus ^22^. Our analysis suggests that those findings may have been driven more by HHV-6 than EBV. However, we cannot rule out EBV reactivation occurring at a different site, which we could not detect using saliva.

Further exploratory analysis found a positive association between HHV-6 shedding and anxiety and depression. HHV-6 has wide tropism, including CNS cells; olfactory astrocytes are a major site of latency and a likely mode of entry into the CNS ^80-83^. Detection of HHV-6 in the Purkinje cells of human post-mortem cerebellar tissue was associated with major depressive disorder (MDD), bipolar disorder, and suicide^81^. Those with MDD also had higher antibody titers against the latent HHV-6B protein SITH-1^83^, which was mechanistically linked to olfactory bulb apoptosis, hypothalamic pituitary adrenal axis activation, and increased corticotropin-releasing hormone^82^. Indeed, it has been reported previously that antibodies against HHV-6 were positively associated with higher cortisol ^84^. While we observed a positive trend between HHV-6 shedding and a higher CAR AUC in the LC group, it did not reach statistical significance (p= 0.43, Supplementary Figure 6E). Taken together, these findings suggest that HHV-6 may play a direct, neuropathological role, though understanding the relationship between HHV-6, stress, and hormone responses requires more work.

This study has several limitations. Firstly, the modest sample size limited the statistical power of some analyses, and we only captured a single period of hormonal and viral fluctuations in each participant. Secondly, medication use was high, especially in the LC group. We accounted for this with strict post-hoc exclusions, but these relied largely on self-report data, supplemented with EMR review where possible. Although we centrifuged our samples to remove cells and calibrated our herpesvirus quantification to human total nucleic acid quantity, it is possible that some herpesvirus DNA originated from latently infected cells. We further mitigated this with a stringent positivity threshold of LLOQ in ≥2 samples per individual.

Our study design used self-collected, unsupplemented saliva, which maximized convenience for participants and the breadth of compatible assays. However, the lack of a specialized storage buffer meant that reliable, comparative quantification of herpesvirus mRNA, which would have offered a conclusive method to confirm herpesvirus reactivation and further probe potentially relevant latency programs, was not possible. Indeed, it is possible that latent EBV might contribute to LC pathology, for example through reprogramming B-cells and driving autoimmunity^85^. Future, longitudinal studies utilizing transcriptomic approaches are needed to more comprehensively assess the latency and reactivation stages of all HHVs, including HHV7 and 8, over the course of LC development, and their associations with LC pathology.

Our results suggest distinct roles for EBV and HHV-6 in LC. For EBV, the cumulative evidence suggests that reactivation before, during or shortly after acute SARS-CoV-2 infection may trigger pathological events that persist beyond acute infection. In contrast, persistent reactivation of HHV-6 in LC may actively contribute to pathology. However, whether HHV-6 reactivation is a response to, or a trigger of, increased symptom severity remains to be investigated. Off-label use of anti-herpes antivirals has been reported among those with LC ^86,87^, but the evidence base supporting their use is scarce, with only one clinical trial currently ongoing ^88^. Furthermore, no drugs have been approved specifically for treating EBV or HHV-6 in any setting, and there is limited-to-no evidence supporting the effectiveness of commonly prescribed nucleoside analogs such as valacyclovir ^89,90^. Development of effective antivirals, vaccines, and monoclonal antibodies for EBV and HHV-6 is needed to examine the role of these persistent herpesviruses in LC and other post-acute infection syndromes.

## Supplementary Material

Supplement 1

## Figures and Tables

**Table 1:** Summary statistics from participant health questionnaires.

Variable	N LC /Control	N pairs tested	LC (mean, SD)	Control (mean, SD)	adj. p- value	Effect size, r
EQ-5D-5L, USA-indexed score	45/45	45	0.6 ± 0.3	0.9 ± 0.1	**<0.001**	−0.87
FSS-7 mean	45/45	45	5.5 ± 1.6	1.7 ± 0.9	**<0.001**	0.79
PHQ-2 sum	45/45	45	2.1 ± 1.4	0.5 ± 0.9	**<0.001**	0.19
GAD-7 sum	45/45	45	7.6 ± 5.5	3 ± 3.1	**<0.001**	0.52
S-LANSS sum	27/0	0	8 ± 5.7	-	-	-
ESS sum	45/45	45	7.2 ± 4.3	3.7 ± 2.9	**<0.001**	0.36
PROMIS Participation T-score	45/45	45	36.9 ± 7	59.3 ± 7.2	**<0.001**	−0.86
NQ-Cognitive T-score	45/45	45	38.8 ± 8	54.5 ± 7.4	**<0.001**	−0.85
DSQ-PEM sum	45/45	45	28.8 ± 20.5	0.3 ± 0.9	**<0.001**	0.87
MRC dyspnea	45/45	45	4.4 ± 2.1	6.8 ± 2.1	**<0.001**	−0.62
SQS	45/45	45	2.4 ± 1	1.1 ± 0.3	**<0.001**	0.38
GSQ-30 sum	44/44	43	48.4 ± 21	6.4 ± 5.9	**<0.001**	0.87
eq5 VAS	45/45	45	49.9 ± 20.3	86.2 ± 8.4	**<0.001**	−0.87
Fatigue VAS	45/45	45	63.4 ± 18.6	14.6 ± 15.6	**<0.001**	0.79
Breathlessness VAS	45/45	45	35.8 ± 22.9	5.8 ± 15.7	**<0.001**	0.6

Differences were evaluated by Wilcoxon Signed-rank tests. Benjamini-Hochberg False Discovery Rate (FDR) adjusted (adj.) p <0.05 was considered significant LC= Long COVID.

## Data Availability

The pre-specified rubric used for symptom survey encoding, the qPCR analysis pipeline, statistical model results, and relevant de-identified data included in our analyses will be included as supplementary files and/or deposited on GitHub upon publication of the peer-reviewed manuscript.
