# Supplementary material for: Analysis Of Salivary Herpesviruses Reveals Associations Between HHV-6 And Long COVID Severity
Source: medRxiv. 2026 May 21:2026.05.19.26353495. Preprint. [Version 1] doi: 10.64898/2026.05.19.26353495 (PMC13228757; doi:10.64898/2026.05.19.26353495)
Supplement: Supplementary file 1 [file NIHPP2026.05.19.26353495v1-supplement-1.pdf]

medRxiv preprint doi: https://doi.org/10.64898/2026.05.19.26353495; this version posted May 21, 2026. The copyright holder for this preprint (which was not certified by peer review) is the author/funder, who has granted medRxiv a license to display the preprint in perpetuity.
It is made available under a CC-BY 4.0 International license .

Supplementary Figures and Tables:

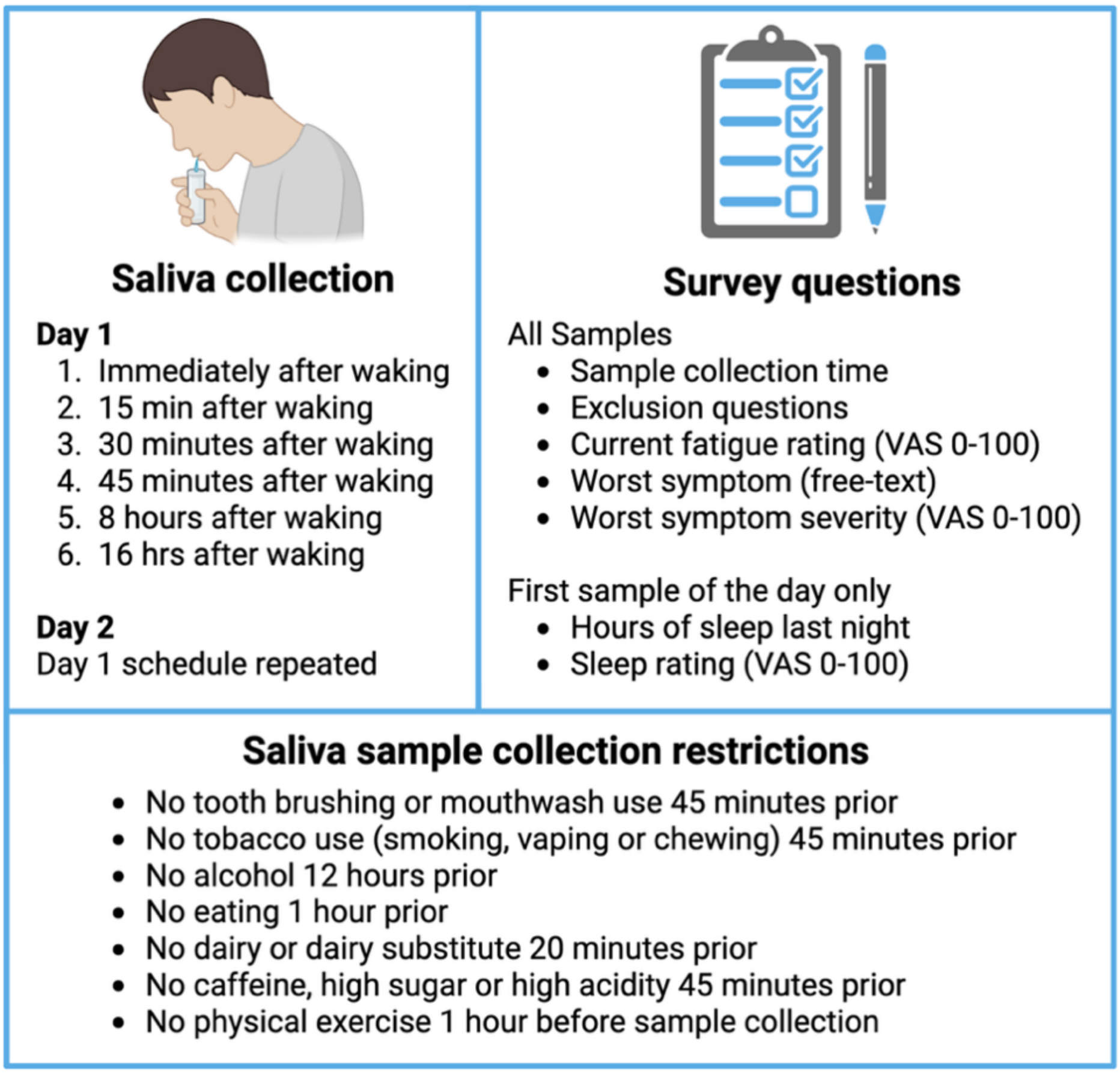

Supplementary Figure 1: Saliva and daily sample survey collection schedule and restrictions. *As far as possible, surveys were collected at the same time as the saliva samples.*

medRxiv preprint doi: https://doi.org/10.64898/2026.05.19.26353495; this version posted May 21, 2026. The copyright holder for this preprint (which was not certified by peer review) is the author/funder, who has granted medRxiv a license to display the preprint in perpetuity.
It is made available under a CC-BY 4.0 International license .

571 ***Supplementary Table 1: Primers and probes used in this study.***

**Target**	**Forward 5'-3' (final nM)**	**Reverse 5'-3' (final nM)**	**Probe 5'-3' (final nM)**	**Source**
**Herpesvirus multiplex**				
**EBV** (BNRF1 gene)	GGAACCTGGTC ATCCTTTGC (300 nM)	ACGTGCATGGAC CGGTTAAT (300 nM)	(FAM)-CGCAGGCACTCGTACTGCTC GCT-(IABKFQ) (200 nM)	[91]
**HCMV** (glycoprotein B gene)	TGAAGCGCCGC ATTGA (400 nM)	TGGCCCGTAGGT CATCCA (400 nM)	(HEX)-TCTGCATGAAGGTCTTTGCCC AGTACATCC-(IABKFQ) (200 nM)	Modified from [92]
**HSV** (1&2, (polymerase gene-coding region)	AGAGGGACATC CAGGACTTTG (200 nM)	GAGCTTGTAATAC ACCGTCAGG (200 nM)	(CY5)-CCGCCGAACTGAGCAGACAC CCGCGC-(IABKFQ) (100 nM)	Modified from [93]
**HHV-6** (pan A/B U67-ORF gene)	CAAAGCCAAATT ATCCAGAGCG (400 nM)	CGCTAGGTTGAG GATGATCGA (400 nM)	(ROX)-CACCAGACGTCACACCCGAA GGAAT-(IABRQSP) (200 nM)	[94]
**SARS-CoV-2 / RNAseP dual-plex**				
**Human** (RNase P subunit 30 gene)	AGATTTGGACCT GCGAGCG (150 nM)	GAGCGGCTGTCT CCACAAGT (150 nM)	(CY5)-TTCTGACCTGAAGGCTCTGCG CG-(BHQ1) (200 nM)	[95]
**SARS-CoV-2** (N1 gene)	GACCCCAAAAT CAGCGAAAT (500 nM)	TCTGGTTACTGC CAGTTGAATCTG (500 nM)	(FAM)-ACCCCGCATTACGTTTGGTGG ACC-(BHQ1) (125 nM)	[95]

medRxiv preprint doi: https://doi.org/10.64898/2026.05.19.26353495; this version posted May 21, 2026. The copyright holder for this preprint (which was not certified by peer review) is the author/funder, who has granted medRxiv a license to display the preprint in perpetuity.
It is made available under a CC-BY 4.0 International license .

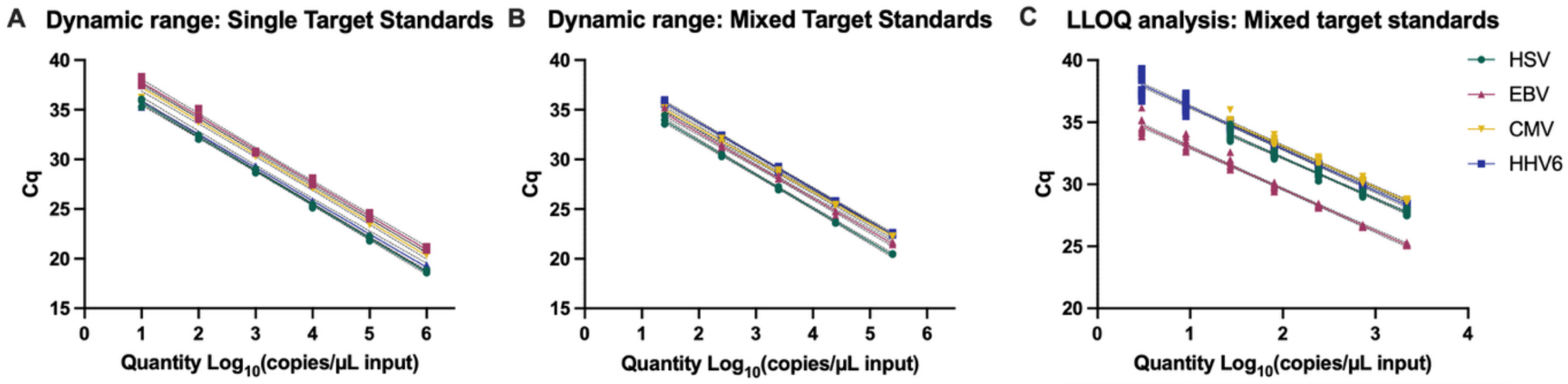

Single target	EBV	CMV	HSV	HHV6
Efficiency	96.2%	98.8%	97.3%	98.1%
R-Squared	0.999	0.994	0.996	0.997

Mixed target	EBV	CMV	HSV	HHV6
Efficiency	97.4%	101.6%	105.2%	100.1%
R-Squared	0.999	0.994	0.996	0.997

Mixed target	EBV	CMV	HSV	HHV6
Efficiency	100.9%	98.1%	100.2%	97.0%
R-Squared	0.985	0.980	0.962	0.984

Supplementary Figure 2: Representative standard curves for EBV, CMV, HSV and HHV-6 detection using quantitative genomic DNA standards. *Amplification was carried out with 1:10 serial dilutions of the quantitative gDNA for either one target of interest (**A**, single target, 200,000-2 copies/reaction), or a 1:1:1:1 mix of all targets (**B**, mixed target, 250,000-25 copies/reaction). Lower limits of quantification (LLOQ) were calculated (**C**, mixed-target, 2187-3 copies/reaction). LLOQ were 3 copies/µL input for EBV and HHV-6 and 27 copies/µL input for CMV and HSV. Efficiencies and* R^2 *values from the simple linear regression (with 95% confidence interval, dashed lines) for each curve are shown in a panel below each experiment.*

medRxiv preprint doi: https://doi.org/10.64898/2026.05.19.26353495; this version posted May 21, 2026. The copyright holder for this preprint (which was not certified by peer review) is the author/funder, who has granted medRxiv a license to display the preprint in perpetuity.
It is made available under a CC-BY 4.0 International license .

Supplementary Table 2: Baseline demographic and clinical characteristics of age-sex-matched Long COVID and Control participants.

**Demographics**	**Control**	**Long COVID**	**SMD**	**Δ**
Age, median (IQR)	36 ± 12	37 ± 13	**0.366**	0.53
BMI, median (IQR)	25 ± 6.4	23.5 ± 8.1	-0.071	-0.66
% Female	73.3% (33/45)	73.3% (33/45)	0	0
Race: Asian	13.3% (6/45)	8.9% (4/45)	-0.142	-4.4
Race: Black or African American	0% (0/45)	2.2% (1/45)	-	-
Race: Other	6.7% (3/45)	17.8% (8/45)	**0.344**	11.1
Race: White, other	2.2% (1/45)	2.2% (1/45)	0	0
Race: White, Hispanic	0% (0/45)	8.9% (4/45)	-	-
Race: White, non-Hispanic	77.8% (35/45)	60% (27/45)	**-0.391**	-17.8
**COVID-19 history (self-report)**				
Previously had COVID-19	60% (27/45)	100% (45/45)	**1.155**	40
Days since first COVID-19, median (IQR)	877 ± 433.5	907 ± 776	0.186	77.27
Days since last COVID-19, median (IQR)	294 ± 263	270 ± 293	**-0.364**	-77.25
Positive test method: PCR	44.4% (12/27)	53.3% (24/45)	-	8.9
Positive test method: Antibody	0% (0/27)	8.9% (4/45)	-	-
Positive test method: Antigen	51.9% (14/27)	17.8% (8/45)	-	-34.1
Positive test method: None	3.7% (1/27)	20% (9/45)	-	16.3
Previously vaccinated against COVID-19	100% (45/45)	100% (45/45)	0	0
Days since COVID vaccine, median (IQR)	293 ± 531	280 ± 391	-0.072	-32.47
**Reported medication use (last 3 months)**				
Anti-anxiety treatment	2.2% (1/45)	24.4% (11/45)	**0.692**	22.2
Anti-hypertensives	6.7% (3/45)	4.4% (2/45)	-0.097	-2.2
Anti-platelet medication	0% (0/45)	8.9% (4/45)	**0.442**	8.9
Anti-psychotics	0% (0/45)	0% (0/45)	**-**	0
Antibiotics	6.7% (3/45)	2.2% (1/45)	**-0.217**	-4.4
Antidepressants	8.9% (4/45)	22.2% (10/45)	**0.374**	13.3
Antivirals	4.4% (2/45)	6.7% (3/45)	0.097	2.2
Benzodiazepine	4.4% (2/45)	4.4% (2/45)	0	0
Blood thinners	2.2% (1/45)	13.3% (6/45)	**0.424**	11.1
Coenzyme Q10 or N-Acetyl L-Cysteine	4.4% (2/45)	51.1% (23/45)	**1.221**	46.7
Diabetes treatment	2.2% (1/45)	4.4% (2/45)	0.124	2.2
Hormonal medications	20% (9/45)	22.2% (10/45)	0.054	2.2
Steroids (inhaled/nasal)	0% (0/45)	0% (0/45)	**-**	0
NSAIDs	33.3% (15/45)	33.3% (15/45)	0	0
Nattokinase, serrapeptase or lumbrokinase	2.2% (1/45)	26.7% (12/45)	**0.742**	24.4
**Medical history (self-reported, past or current)**				
Anxiety	20% (9/45)	55.6% (25/45)	**0.788**	35.6
Asthma	11.1% (5/45)	24.4% (11/45)	**0.354**	13.3
Auto-immune disease	0% (0/45)	0% (0/45)	-	-
Cancer	4.4% (2/45)	2.2% (1/45)	-0.124	-2.2
Chronic kidney disease	0% (0/45)	0% (0/45)	-	-
Chronic liver disease	0% (0/45)	0% (0/45)	-	-

medRxiv preprint doi: https://doi.org/10.64898/2026.05.19.26353495; this version posted May 21, 2026. The copyright holder for this preprint (which was not certified by peer review) is the author/funder, who has granted medRxiv a license to display the preprint in perpetuity.
It is made available under a CC-BY 4.0 International license .

Chronic obstructive pulmonary disease	0% (0/45)	0% (0/45)	-	-
Depression	20% (9/45)	35.6% (16/45)	**0.353**	15.6
Diabetes	0% (0/45)	0% (0/45)	-	-
Eating disorder	6.7% (3/45)	2.2% (1/45)	**-0.217**	-4.4
Hormonal disorder	2.2% (1/45)	6.7% (3/45)	**0.217**	4.4
Hypertension	4.4% (2/45)	6.7% (3/45)	0.097	2.2
Obesity	8.9% (4/45)	6.7% (3/45)	-0.083	-2.2
Other chronic lung disease	0% (0/45)	4.4% (2/45)	**0.305**	4.4
Other neurological disease	0% (0/45)	2.2% (1/45)	**0.213**	2.2
Other psychiatric disease	4.4% (2/45)	4.4% (2/45)	0	0
Spinal cord injury	0% (0/45)	0% (0/45)	-	-
Stroke	0% (0/45)	0% (0/45)	-	-
Other comorbidities	11.1% (5/45)	35.6% (16/45)	**0.604**	24.4

SMD= Standardized mean difference between groups (mean difference / pooled SD of the matched pair differences for continuous variables; difference in proportions / pooled binomial SD for categorical variables, reported per category). SMD ≥0.20 is indicative of meaningful imbalance and is shown in bold.

Δ= Absolute difference between groups (mean difference for continuous variables; percentage-point difference for categorical variables).

medRxiv preprint doi: https://doi.org/10.64898/2026.05.19.26353495; this version posted May 21, 2026. The copyright holder for this preprint (which was not certified by peer review) is the author/funder, who has granted medRxiv a license to display the preprint in perpetuity.
It is made available under a CC-BY 4.0 International license .

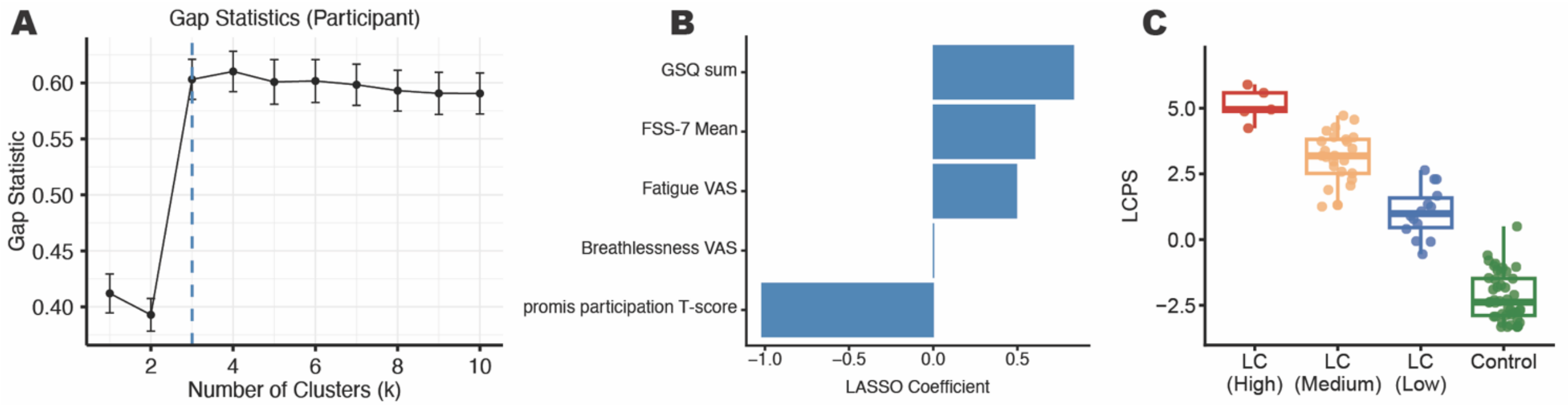

Supplementary Figure 3: A. *Gap statistics for determining the optimal number of clusters of participants for the GSQ-30 symptom clustering shown in* ***Figure 2A****.* ***B.*** *LASSO coefficients of the non-zero contributors to the LCPS model (GSQ Sum= Sum of General Symptom Questionnaire 30, FSS-7 Mean= mean fatigue severity scale score, VAS= visual analog scale for fatigue and breathlessness, promis participation T-score= Adult PROMIS Ability to Participate in Social Roles and Activities short form.* ***C.*** *LCPS for each participant, plotted according to their GSQ-30 severity cluster****.***

medRxiv preprint doi: https://doi.org/10.64898/2026.05.19.26353495; this version posted May 21, 2026. The copyright holder for this preprint (which was not certified by peer review) is the author/funder, who has granted medRxiv a license to display the preprint in perpetuity.
It is made available under a CC-BY 4.0 International license .

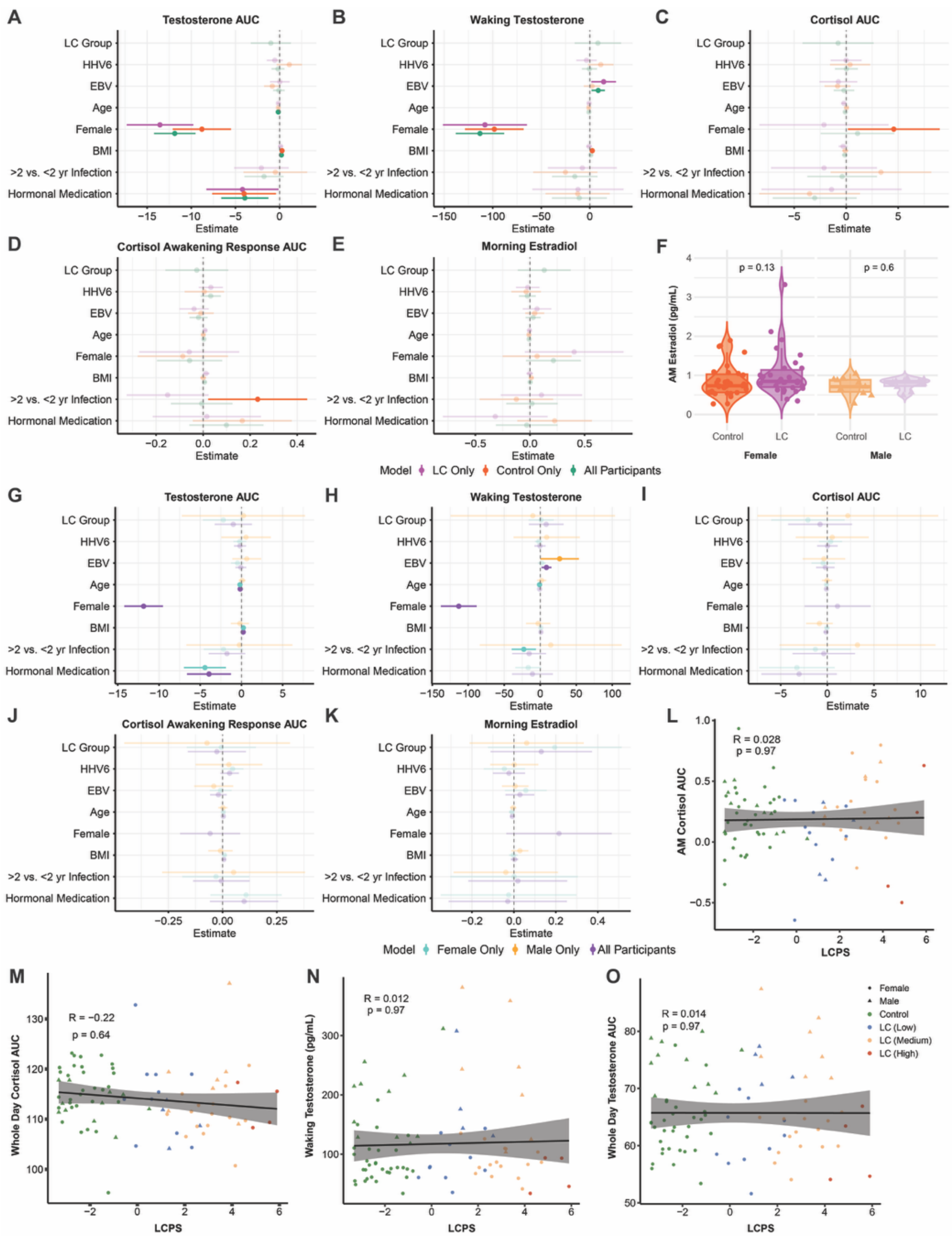

Supplementary Figure 4: Relationships between salivary hormones, demographics, and Long COVID severity. *Forest plots showing the association between Whole Day Testosterone AUC (**A,G**), Waking Testosterone (**B,H**), Whole Day Cortisol AUC (**C,I**), Cortisol Awakening Response AUC (**D,J**) and Morning Estradiol (**E,K**) and Long COVID status, EBV and HHV-6 mean log (viral copies) +1, adjusted for age, sex, hormonal medication, BMI, and time from first COVID infection, stratified by Long COVID status (**A-E**) and sex (**G-K**). Regression coefficients are reported with 95% confidence intervals, and those in bold are associations with nominal p<0.05.* ***F.*** ***Comparison of single timepoint morning salivary Estradiol levels by group.*** *n=30 (LC Female), n=10 (LC Male), n=31 (Control Female), and n=11 (Control Male). Central lines indicate the group median, top and bottom lines indicate the 75th and 25th percentiles, respectively, the whiskers represent 1.5 x the interquartile range. Each dot represents one individual. Statistical significance of the difference in median values was determined using Wilcoxon ranked-sum test.* ***L-O.*** *Correlation analysis between Long COVID propensity score and Cortisol Awakening Response AUC (**L**), Whole Day Cortisol AUC (**M**), Waking Testosterone (**N**), or Whole Day Testosterone AUC (**O**). Spearman coefficients (R) are shown, with significance determined by linear regression adjusting age, sex and BMI. p <0.05 was considered significant. Benjamini-Hochberg False Discovery Rate was used to correct for multiple comparisons. The black line depicts linear regression, and the shading shows the 95% CIs. Each dot represents one individual. n = 84 (41 LC, 43 control).*

medRxiv preprint doi: https://doi.org/10.64898/2026.05.19.26353495; this version posted May 21, 2026. The copyright holder for this preprint (which was not certified by peer review) is the author/funder, who has granted medRxiv a license to display the preprint in perpetuity.
It is made available under a CC-BY 4.0 International license .

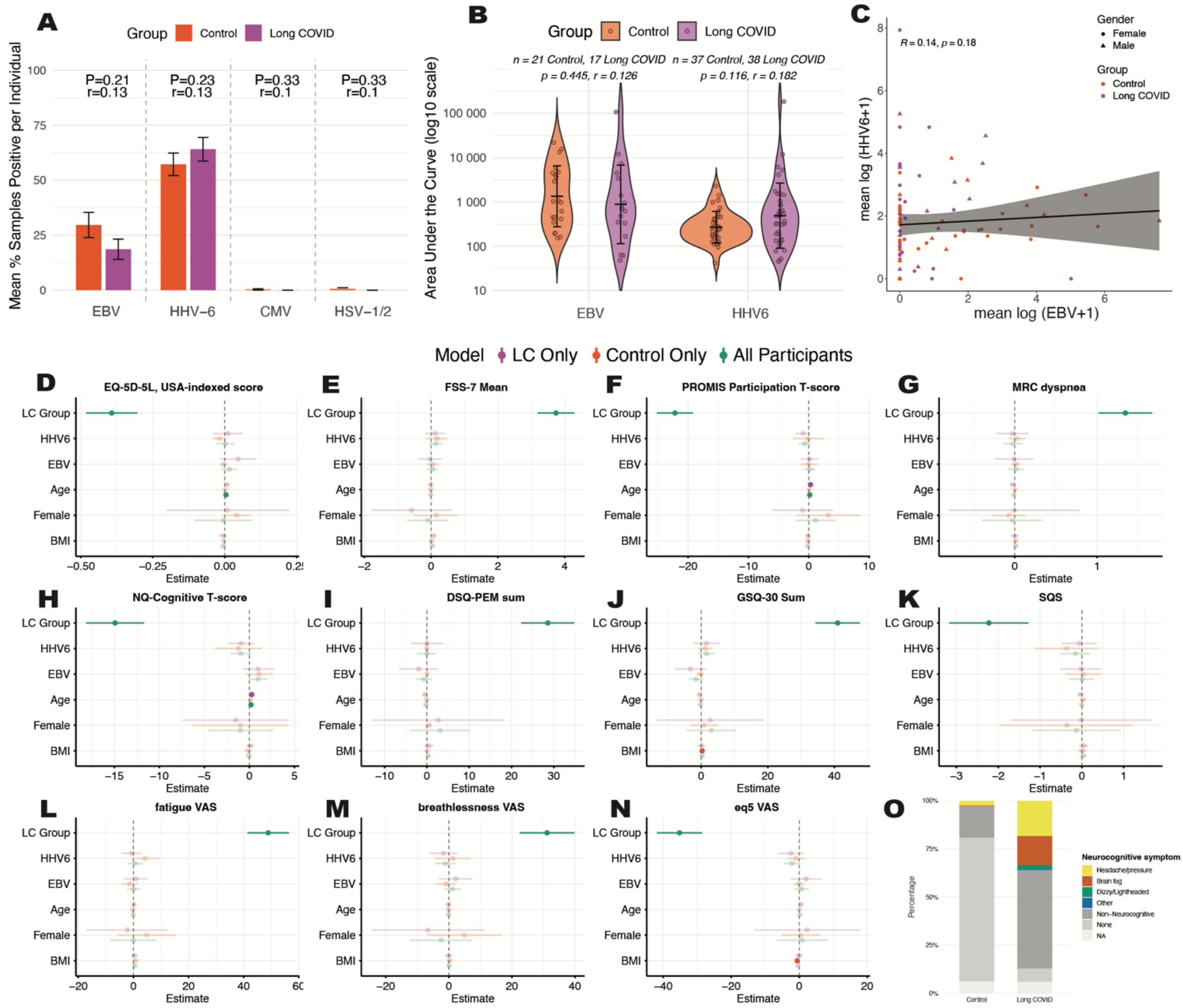

Supplementary Figure 5: Relationships between detection of salivary herpesviruses, demographics, and Long COVID severity. A. *Proportion of samples per participant positive for each virus. Statistical differences tested using Fisher's Exact test, OR= Odd's Ratio, P<0.05 is significant.* ***B.*** *Comparison of area under the curve of viral DNA copies, averaged over 2 days, between participants with Long COVID and controls. Mean and SD are shown. Statistical differences tested using Wilcoxon rank-sum test, effect size reported as r (Z / √N), P<0.05 is significant.* ***C.*** *Spearman correlation between mean log (HHV-6 +1) and log (EBV+1) viral copies in saliva. P<0.05 is significant.* ***D-N.*** *Forest plots showing the association between health questionnaire outcomes and Long COVID (LC) status, EBV and HHV-6 mean log (viral copies) +1, adjusted for age, sex and BMI, and stratified by Long COVID status: quality of life (**D, N**), fatigue (**E, L**), ability to participate in daily activities (**F**), dyspnea (**G, M**), perceived cognitive ability (**H**), post-exertional malaise (**I**), general somatic symptom burden (**J**) and sleep quality (**K**). Regression coefficients are reported with 95% confidence intervals and those in bold are associations with p<0.05.* ***O.*** *Incidence of neurocognitive (NC) symptom being reported as the 'worst symptom' by participants at a given timepoint, separated by subtypes (NA = survey question not answered).*

medRxiv preprint doi: https://doi.org/10.64898/2026.05.19.26353495; this version posted May 21, 2026. The copyright holder for this preprint (which was not certified by peer review) is the author/funder, who has granted medRxiv a license to display the preprint in perpetuity.
It is made available under a CC-BY 4.0 International license .

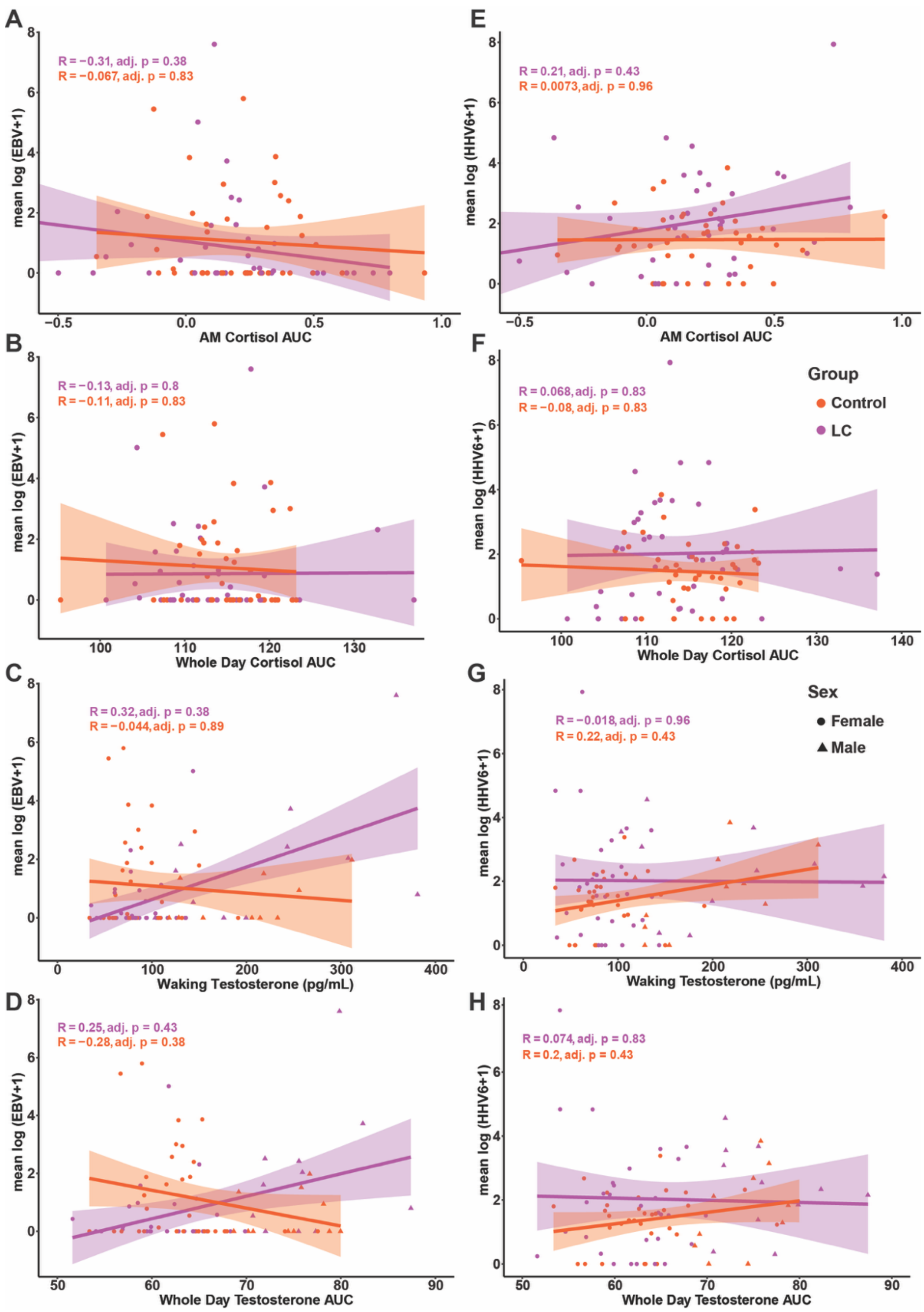

Supplementary Figure 6: Relationships between salivary hormones and viral shedding. A-D. *Correlation analysis between salivary EBV DNA copies and Cortisol Awakening Response AUC (**A**), Whole Day Cortisol AUC (**B**), Waking Testosterone (**C**), and Whole Day Testosterone AUC (**D**) separated by group.* ***E-H.*** *Correlation analysis between salivary HHV-6 DNA copies and Cortisol Awakening Response AUC (**E**), Whole Day Cortisol AUC (**F**), Waking Testosterone (**G**), and Whole Day Testosterone AUC (**H**) separated by group. Spearman coefficients (R) are shown, with significance determined by linear regression. Benjamini-Hochberg False Discovery Rate (FDR) was used to correct for multiple comparisons. p <0.05 was considered significant. The dark, solid lines depict linear regression, and the shading shows the 95% CIs. Each dot represents one individual. n=41 (LC) and n=43 (Control).*

medRxiv preprint doi: https://doi.org/10.64898/2026.05.19.26353495; this version posted May 21, 2026. The copyright holder for this preprint (which was not certified by peer review) is the author/funder, who has granted medRxiv a license to display the preprint in perpetuity.
It is made available under a CC-BY 4.0 International license .

Supplementary methods:

Participant recruitment

LC and Control participants were recruited from the New York City area via social media posts and flyers posted throughout the Mount Sinai Health System. Participants with LC were also recruited from the Cohen Center for Recovery from Complex Chronic Illness and Center for Post COVID-19 Care at the Icahn School of Medicine at Mount Sinai, including individuals that have previously attended the clinic/center. If the physician reviewing the patient identified that they met the inclusion/exclusion criteria, with the patient's permission, they connected patients with the study coordinator for enrollment. Control participants were also recruited via emails to an existing database of individuals with past COVID-19 infection who received clinical home monitoring from the Abilities Research Center team during acute COVID-19 infection.

Timepoint survey 'worst symptom' encoding

The free-text answer to the question "Excluding fatigue, what is the worst symptom you are experiencing at this exact moment" was encoded into the end-organ systems using pre-specified criteria that had been agreed upon by two physicians (**Supplementary Document 1**). Encoding was completed twice for each survey, by two independent encoders. Final encoding was independently reviewed by a physician, who settled discrepancies and ambiguous answers, and any answer that was too ambiguous to be categorized was encoded as "Other." Additional encoding of neurocognitive symptom sub-types was completed in the same fashion. Sub-types were categorized as either head pain/pressure (included references to headaches, migraines, pressure in head), dizziness, brain fog (included all references to "brain fog", issues with reasoning, memory, attention, and speed of thought). The term brain fog was selected as the umbrella term as it was the most frequent phrasing chosen by participants and is consistent with usage in previous studies[96].

Hormone assay details and batch correction

For cortisol, assay sensitivity was 0.007 μg/dL, assay range was 0.012 – 3.0 μg/dL and Intra-assay coefficients of variation (CVs) were 4.6 – 6% [56]. For testosterone, assay sensitivity was 1.0 pg/mL, assay range was 6.1 – 600 pg/mL and Intra-assay CVs were 4.6 – 9.85% [57]. For

medRxiv preprint doi: https://doi.org/10.64898/2026.05.19.26353495; this version posted May 21, 2026. The copyright holder for this preprint (which was not certified by peer review) is the author/funder, who has granted medRxiv a license to display the preprint in perpetuity.
It is made available under a CC-BY 4.0 International license .

estradiol, assay sensitivity was 0.1 pg/mL, assay range was 1 - 32 pg/mL and Intra-assay CVs were 7.13 –7.45% [58]. Analysis of saliva for cortisol and testosterone was conducted in three batches with internal controls in each shipment to correct for kit batch effects. Estradiol analysis was conducted in a single batch.

To integrate analysis across batches, repeated samples from multiple timepoints for 7 different study participants across groups (LC and Control) were measured between Batch 1 and 2 (n=57 total) and for 5 different study participants across groups were measured between Batch 2 and 3 (n=53 total). A linear model was fit between Batch 1 and 2 and between Batch 2 and 3. No adjustments were made for demographic covariates because study participants were age- and sex-matched. A multiplicative coefficient (the slope of the linear regression line) was used to correct data, with Batch 2 as the reference. For testosterone, the intercepts against batch 2 were 3.93 and 10.47 for batch 1 and 3, respectively, and these were added to each value during correction. For cortisol, the intercepts were <0.01, so no additional additive correction was used.

Standard curve analysis for qPCR

Standard curves analyses were done using simple linear regression with GraphPad Prism (Version 10.6.1). Efficiency was established using standard curves across a dynamic range of 10^1-10^6 copies/reaction conducted in triplicate (**Supplementary Figure 2A-B**). Lower limit of quantification (LLOQ) for each target was established with 12 replicates for each dilution over a range of 3-2187 copies/reaction (**Supplementary Figure 2C**). LLOQ was the lowest value which could be included in analysis of curves which meet the following criteria: amplification of 11/12 replicates with a Cq SD <1, efficiency ($10^{(-1/slope)-1}$) of 95%-105%, $R^2 > 0.9800$, and passing the replicates test for lack of fit ($p>0.05$) An individual was considered positive if a given virus was detected in ≥2 of their samples above the LLOQ.

Automated qPCR pipeline protocol

The pipeline itself and supporting files will be deposited in GitHub upon publication. Within the pipeline, data were first extracted from the raw .csv files exported from the BioRad CFX96 qPCR machines, and sample IDs and targets automatically assigned according to user-supplied plate maps and fluorescent channel target assignments. Runs were then QC'd to flag negative controls (NTC and NEC) with amplification or positive controls without

medRxiv preprint doi: https://doi.org/10.64898/2026.05.19.26353495; this version posted May 21, 2026. The copyright holder for this preprint (which was not certified by peer review) is the author/funder, who has granted medRxiv a license to display the preprint in perpetuity.
It is made available under a CC-BY 4.0 International license .

amplification, and that all samples had amplification for an internal control, here, RNAseP (adjustment for RNAseP or another internal control is a toggleable feature of the pipeline).

Next, the mean and SD for the Cycle of quantitation (Cq) and the Starting quantity (SQ), which were interpolated from the standard curve using the BioRad CFX96 software prior to export, were calculated for each duplicate. Samples were then flagged as "Inconclusive" where they: contained replicates which amplified with Cq <10, had only one replicate which amplified and with a Cq >35, or had more than 1 replicate with a Cq SD ≤2. Finally, the mean SQ for each herpesvirus was normalized according to total input nucleic acid by multiplying by a correction factor, which was calculated for each sample by dividing the mean sample RNaseP Cq by the global median RNAseP Cq (another target can be selected in place of RNaseP by the user).

The outputs are: a combined file of all collated raw data with sample IDs and targets assigned, a clean file following QC with technical replicates (and inter-plate replicates if repeated) collapsed into a single mean and corrected for RNAseP (or another internal control if enabled), and an exclusions sheet listing Inconclusive samples which were excluded and flagged for repeat, and the positive/negative controls for each run, with problematic controls flagged. Where inconclusive samples are re-run, and added to the data, the pipeline can weigh collective results from the same ID and make a consensus call to resolve inconclusive data (averaging the remaining included data), those points which were discarded are also included in the exclusion output but marked as not requiring repeat.
